# The Hospital, Nursing, and Midwifery Exhibition

**Published:** 1924-06

**Authors:** 


### THE HOSPITAL EXHIBITION.

The Hospital, Nursing, and Midwifery Exhibition was opened at the Central Hall, Westminster, on Monday, May 19, by Princess Marie Louise, who was received by Mr. Schofield, Mr. Eason, C.B., Colonel Blackham, C.B., and Mr. Godfrey Hamilton. After a short speech of welcome by Mr. Schofield, Her Highness wished the exhibition every success; it was, she said, of a much-needed character. A tour of the exhibits was then made. Doctors and nurses and all those interested in public health work found the exhibition, which remained open till the end of the week, well up to its usual level—indeed, the display seemed more extensive than ever. There were also the customary lectures in connection with the exhibition which were deservedly well attended. The speakers included Professor Louise McIlroy, of the Gynæcological Unit of the Royal Free Hospital, who dealt with some problems of infant mortality; Miss Dane, matron of Queen Charlotte's Hospital; Miss A. Masters, superintendent of the Paddington and St. Marylebone Nursing Association; and Dr. Elizabeth Sloan Chesser, whose paper was devoted to " The Psychological Aspect of Sick Nursing." Colonel Blackham spoke on " Tropical Nursing," Dr. Sibley on " General Principles of the Treatment of Diseases of the Skin," Dr. Salmond on " Radiology," and Mr. Sanderson Hall on " The Insurance Consultant's Value to the Nursing Profession."

